# Psychosis in autism spectrum disorder: a clinical review

**DOI:** 10.1192/j.eurpsy.2023.1589

**Published:** 2023-07-19

**Authors:** D. R. Gomes, F. Silveira, R. Freitas

**Affiliations:** Departamento de Psiquiatria e Saúde Mental, Hospital do Espírito Santo de Évora, Évora, Portugal

## Abstract

**Introduction:**

Autism spectrum disorder (ASD) is a neurodevelopmental disorder characterized by frequent comorbidity including mood, anxiety and psychotic disorders. Psychiatric comorbidity in ASD has been associated with poor prognosis.

**Objectives:**

To summarize clinical data regarding the relationship between autism spectrum disorder and psychosis comorbidity, namely its epidemiology, diagnosis, treatment and prognosis.

**Methods:**

We conducted a non-systematic review of the literature relevant to the topic published in the PubMed database. Articles were selected based on title and abstract review.

**Results:**

Epidemiological studies report significant rates of comorbidity between ASD and psychosis. According to a recent systematic review, prevalence of non-affective psychosis in ASD has been estimated at 9,56%, despite heterogeneity across included studies.

The differential diagnosis of psychosis in a patient with ASD is frequently a challenge and depends on the severity of intellectual and language impairment, medical comorbidities (including epilepsy and associated pharmacological iatrogenic factors), psychiatric comorbidities and substance use. Conversely, establishing the diagnosis of ASD in a patient presenting with psychosis is not always clear, and clinicians must rely on collecting a detailed developmental history.

There are no large controlled studies regarding the treatment of psychosis in this specific patient group, but risperidone and aripiprazole have been used based on efficacy in primary psychotic disorders, as well as efficacy and safety profile in other symptomatic clusters of ASD, namely irritability.

ASD and psychosis comorbidity has been associated with lower response rates to antipsychotic treatment and negative long-term prognosis.

**Conclusions:**

Psychosis is a common and serious comorbidity of ASD, with limited data regarding treatment options. Further research is needed to improve global outcomes.

**Disclosure of Interest:**

None Declared

